# An Energy Efficient Memory Cell for Quantum and Neuromorphic Computing at Low Temperatures

**DOI:** 10.1021/acs.nanolett.4c05855

**Published:** 2025-04-14

**Authors:** Yi Han, Jingxuan Sun, Benjamin Richstein, Andreas Grenmyr, Jin-Hee Bae, Frederic Allibert, Ionut Radu, Detlev Grützmacher, Joachim Knoch, Qing-Tai Zhao

**Affiliations:** † Institute of Semiconductor Nanoelectronics, Peter Grünberg Institute 9 (PGI 9) and JARA-Fundamentals of Future Information Technologies, Forschungszentrum Jülich, Jülich 52428, Germany; ‡ Institute of Semiconductor Electronics, RWTH Aachen University, 52056 Aachen, Germany; § SOITEC, 38190 Bernin, France

**Keywords:** Cryogenic CMOS, Quantum computing, Cryogenic memory, Neuromorphic computing

## Abstract

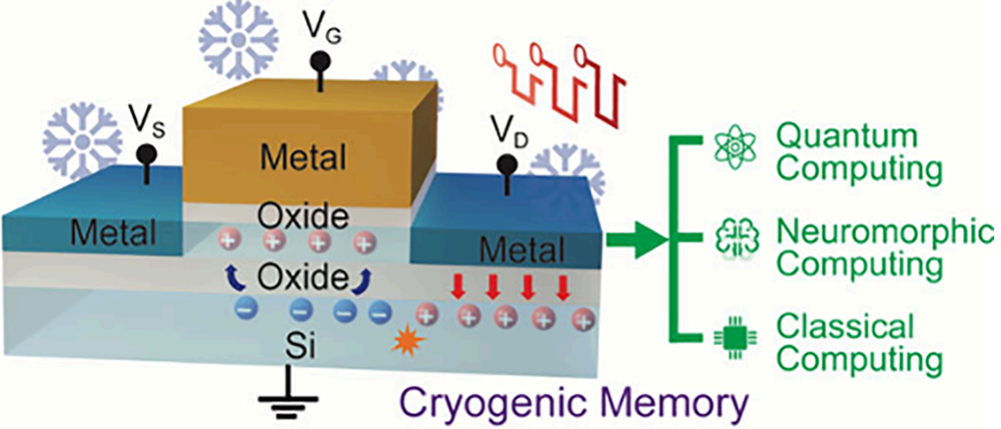

Efficient computing in cryogenic environments, including classical von Neumann, quantum, and neuromorphic systems, is poised to transform big data processing. The quest for high-density, energy-efficient memories continues, with cryogenic memory solutions still unclear. We present a Cryogenic Capacitorless Random Access Memory (C^2^RAM) cell using advanced Si technology, which enhances storage density through its scalability and multistate capability. Remarkably, the C^2^RAM maintains data for over a decade with its extended retention times and offers potential as an artificial synapse. This positions C^2^RAM as an ideal nonvolatile memory candidate for cryogenic computing applications and emerging quantum technologies.

The exponential growth in big data processing has created a pressing need for high-performance computing systems with low power consumption. However, the energy consumption of classical computers based on silicon complementary metal–oxide–semiconductor (CMOS) technology poses a significant challenge for large-scale data centers and clouds. Potential solutions to these challenges have emerged recently and include cryogenic computing as well as novel computing paradigms such as neuromorphic^1−6^ and advanced quantum computing.^7,8^ Interestingly, while in quantum computing, extremely low cryogenic temperatures (≪1 K) are mandatory to preserve quantum coherence necessary for the operation of qubits, cryogenic temperatures are also beneficial for classical von Neumann circuits based on CMOS technology: The strong reduction of the supply voltage of classical CMOS circuits at cryogenic temperatures offers significant power savings beyond the power required for cooling.^9,10^ Moreover, cryogenic neuromorphic computing has recently gained increasing attention.^11,12^ Machine learning can play a pivotal role in implementing and tuning semiconductor spin qubits, realizing error correction decoder and analyzing qubit information.^13−15^ The trends mentioned above open up promising prospects for a universal cryogenic computing (UCC) that combines the benefits of von Neumann, neuromorphic and quantum information processors, especially in the context of large-scale data processing where power efficiency is a critical factor. Apart from transistor devices, the realization of UCC requires also a high density and low power memory. While obvious performance benefits are obtained for the operation of metal-oxide-semiconductor field-effect transistors (MOSFETs) at cryogenic temperatures, no clear path exists to date for storing and retrieving information in an appropriate way.

Attempts have been made to develop cryogenic memories,^16^ including Josephson junction-based memories,^17^ resistance-based memories such as ReRAM,^18^ FeRAM,^19^ MRAM,^20^ and dynamic RAM (DRAM),^21−24^ which are reviewed and benchmarked in ref (16). Among these, DRAM is one of the strongest candidates due to its mature silicon CMOS technology. However, conventional DRAM cells consisting of one transistor and one capacitor are not suitable for high-density integration. To address this issue, capacitorless single transistor DRAM technology (1T-DRAM) exploiting the floating-body (FB) effect in partially depleted silicon-on-insulator (PDSOI) has been proposed.^25^ In 1T-DRAM, charges created by impact ionization or by band-to-band tunneling accumulate at the interface between top Si and the buried oxide (BOX) due to the FB effect. However, this leads to the coexistence of both electrons and holes in the silicon layer. The proximity of the channel and stored charges causes high leakage and hence short retention times. Improving the retention time necessitates either a relatively thick top silicon layer or a high back-gate voltage to effectively separate the channel and storage charges.^26^ While, indeed, the retention time of 1T-DRAM can be increased to a few seconds at cryogenic temperatures,^23^ the use of PDSOI undermines its scalability due to short channel effects (SCE) in a thick SOI layer. Consequently, 1T-DRAM based on PDSOI does not fulfill the low-power and high-density requirements. Note that in ultrathin (<10 nm) fully depleted SOI (FDSOI) as mandatory for device scalability, 1T-DRAM is difficult to be implemented since the supercoupling effect prevents the coexistence of electrons and holes at the two interfaces.^27−30^

To address the above-mentioned issues, we achieved a new Cryogenic Capacitorless RAM (C^2^RAM) cell based on a single FDSOI MOSFET, featuring an ultrathin Si body and an ultrathin BOX (UTBB). Our C^2^RAM benefits from the UTBB SOI structure, allowing for high scalability with suppressed SCE and energy efficiency, as well as extended multistate capability. Unlike PDSOI 1T-DRAM, C^2^RAM does not store charges in the SOI channel layer but instead locates them at the Si substrate-BOX interface, and thus separating them from the channel carriers and the source/drain junctions by the thin BOX layer. This significantly improves the retention time, allowing for nonvolatile memories for cryogenic computing.

The proposed C^2^RAM structure is schematically shown in Figure 1. The memory states are written by applying a voltage V_D_ to the drain. During normal operation, as a low drain voltage V_D_ (Figure 1a) is applied, the energy bands are rather unaffected as indicated in Figure 1b along the dashed line at the BOX/Si substrate interface. In this case the memory is in the “0” state, which can be read at a small V_D_ by sweeping the gate voltage (Figure 1c). If a higher negative V_D_ which is larger than the threshold writing voltage V_TW_ is applied, generating a high enough electric field in the substrate, it causes impact ionization at the substrate/BOX interface as illustrated in Figure 1d and Figure 1e. This leads to the accumulation of holes in the substrate beneath the drain region (Figure 1d) and at the same time, promoting electrons to flow to the region underneath the channel (Figure 1e). At cryogenic temperatures the freeze-out of dopants causes the substrate to be highly resistive. Thus, the charged regions close to the BOX/Si-substrate interface are in a floating state. When the V_D_ is reduced to the small value used for read-out, the majority carriers, i.e. holes, dissolve in the substrate. The electrons underneath the channel region at the BOX/substrate interface, however, are stored. These stored electrons cause the accumulation of holes in the top Si/BOX interface, and thus lower the threshold voltage V_TH_ of the devices. Consequently, the memory state “1” is written. The freeze-out of holes in the substrate prohibits the recombination of the stored electrons, thus preserving a very long retention time. Fully silicided source/drain electrodes and an ultrathin BOX are used in order to increase the capacitive coupling to the Si-substrate underneath drain and thus to reduce the voltage required for writing. The BOX separates the stored charges from the channel and source/drain, yet provides sufficient capacitive coupling to the channel to realize a large memory window (MW). Unlike the PDSOI 1T-DRAM^23^ our device ensures that, the MOSFET remains unaffected by the hot carriers that are typically generated by the high electric fields required for impact ionization in 1T-DRAM. These are the major advantages of the proposed device design. Erasing the written state can be done by applying a positive drain voltage, as shown in Figure 1g. In this case, the stored electrons are annihilated by recombination with holes (Figure 1h). As a result, the state “1” is erased and the memory is reset to state “0” (Figure 1i).

**Figure 1:**
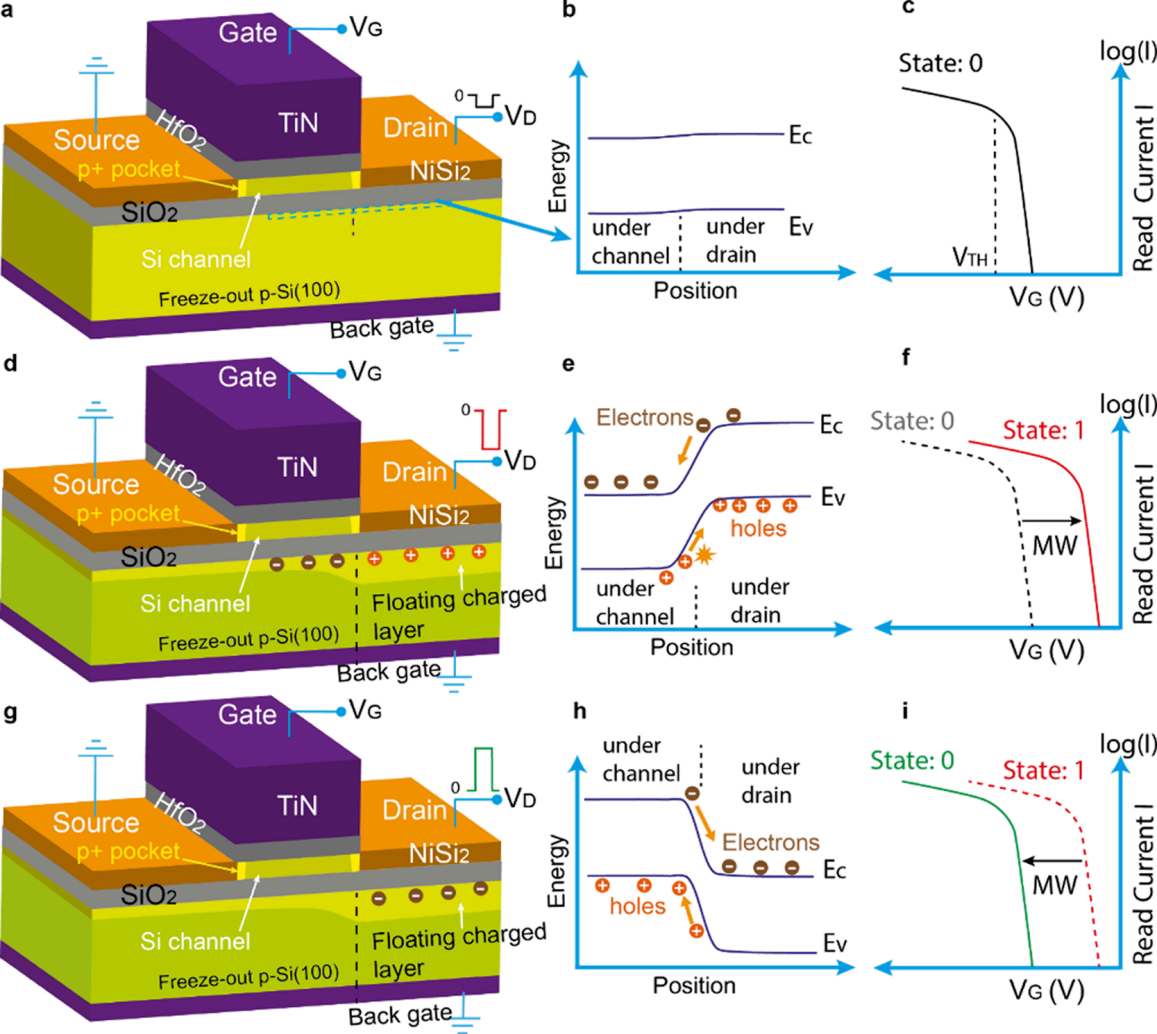
a. Schematic of the device structure operating at a low V_D_ (a negative V_D_). b. Energy band diagram at the substrate surface marked with a dashed line in a. c. The I_D_–V_G_ characteristics showing the “0” state with a higher threshold voltage |V_TH_|. d. Device structure showing the charge distribution in the substrate for state “1” written with a high negative drain voltage V_D_. e. Energy band diagram at the substrate surface showing the impact ionization caused by V_D_. f. The I_D_–V_G_ characteristics showing the “1” state with a smaller threshold voltage |V_TH_|. The memory window (MW) ΔV is defined by the voltage difference between the “0” and the “1” state. g. Device structure showing the charge distribution during erase. h. Energy band diagram at the substrate surface showing the erase process where holes diffuse to the region under the channel to annihilate the electrons accumulated during the write process. i. I_D_–V_G_ characteristics showing the “0” state after erase.

We fabricated C^2^RAM devices on a UTBB SOI wafer. The fabrication process is detailed in the Supporting Information (SI) and Figure S1. The silicon channel layer has a thickness of 9 nm and a BOX thickness of 15 nm, as can be inferred from the cross-sectional transmission electron microscope (TEM) image shown in Figure 2a. The room temperature characteristics of the device are shown in Figure S2. Here, we focus on the memory characteristics measured at 5.5 K. Prior to any writing, we measured the device at a reading drain voltage (V_DR_) of −0.3 V, which served as the initial reference state “0” (represented by the black curves in Figure 2c and f). The voltage configuration is detailed in the SI and Figure 2b,e. As |V_DW_| > |V_TW_| we obtained multiple states by changing the writing voltage V_DW_ from −2.00 to −2.75 V, as displayed in Figure 2c. As we discussed in Figure 1, the memory functionality is caused by impact ionization. For a MOS structure, the impact ionization rate α depends strongly on the electric field:^31^
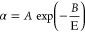
1where *A* and *B* are constants and *E* is the electric field in the Si substrate close to the surface. The impact ionization can be characterized using the substrate currents, as detailed in Figure S4. From eq 1 it is clear that a higher writing voltage increases the impact ionization rate thereby injecting more electrons into the substrate underneath the channel. In turn, this leads to a gradual change of the threshold voltage of the device. The memory window (MW) which is defined as the gate voltage difference with respect to the reference curve of the initial state “0” at a constant I_D_ = 10 nA, increases gradually as |V_DW_| increases (Figure 2d). An MW of ΔV = 0.65 V is achieved at V_DW_ = −2.75 V. Similar behavior is found for the reading current (Figure 2d), thus enabling multiple memory states. This provides the ability to store data in more than one single bit compared to conventional binary memory (0 or 1), allowing a much higher storage density.

**Figure 2:**
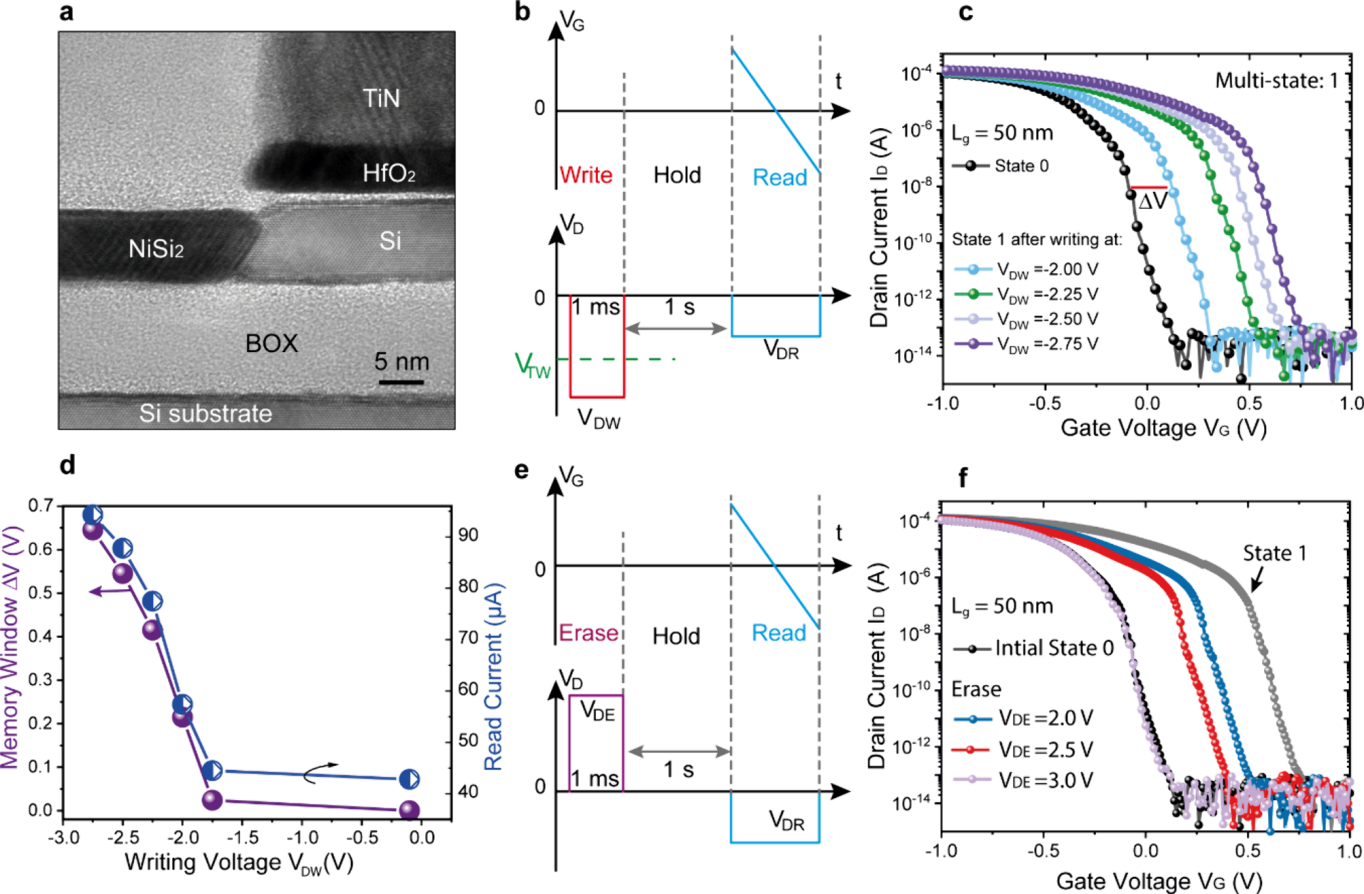
a. Cross-sectional TEM image showing the device structure. b. Voltage configuration for writing, holding and reading state “1” with a negative writing voltage |V_DW_| > |V_TW_|. c. Reading of state “1” after writing, showing multistates. d. Memory window ΔV and reading current as a function of writing voltage V_DW_. e. The voltage configuration for erasing, holding and reading with a positive erasing voltage V_DE_. f. Read-out of the device after erasing of state “1” which is written at V_DW_ = −2.75 V (gray curve). The gate voltage during reading is swept from −1.0 to 1.0 V. For all results shown in Figure 2 the source is grounded during measurements.

To erase the state “1” we apply a positive voltage V_DE_ on the drain as illustrated in Figure 2e. The reading characteristics after erasing with different V_DE_ are shown in Figure 2f. By increasing V_DE_, the I_D_–V_G_ curve shifts to the left. At V_DE_ = 3.0 V the I_D_–V_G_ curve completely overlaps with the initial state “0” reference curve, which is equivalent to a complete erase. Similar to the writing process, the erase program also allows for multiple memory states which could further increase the storage density.

The writing time dependence of a 50 nm gate length device was characterized at 5.5 K, as depicted in Figure 3a. The reading I_D_–V_G_ curves demonstrate that they are nearly identical from 1 μs to 1 ms.

**Figure 3:**
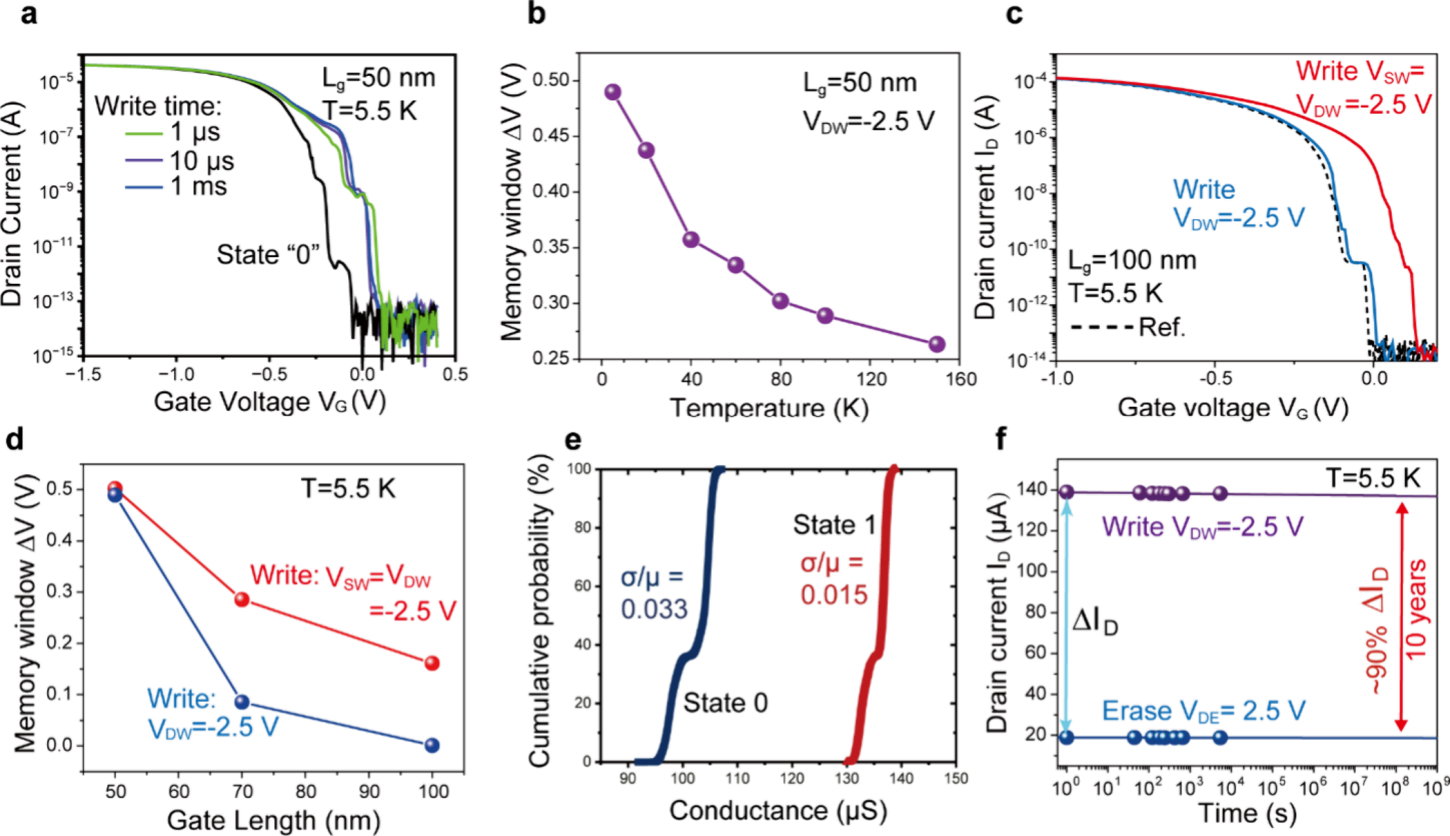
a. Reading of state “1” after writing with the same writing voltage (V_DW_ = −2.5 V) and different writing time. b. Measured memory window ΔV as a function of temperature for devices. c. Comparison of I_D_–V_G_ characteristics for a 100 nm gate length device after writing only at the drain and at both the source and the drain simultaneously. d. Measured memory window ΔV as a function of gate length. Writing simultaneously at both sides increases the memory window for longer gate length devices. e. Cumulative probability distribution of state 0 (before writing at −2.5 V) and 1 (after writing at −2.5 V) showing very small variation. f. Measured reading drain current I_D_ as a function of hold time, showing an exceptionally high retention time. Here, ΔI_D_ represents the disparity between the reading current after writing in 1 s and the reading current after erase in 1 s.

It is important to note that the electrons injected underneath the channel can be stored due to the high resistive and floating substrate at cryogenic temperature. As such, the memory functionality strongly depends on the temperature, as indicated in Figure 3b and further supported by Figure S5. At higher temperatures the conductance of the substrate increases because of a reduced carrier freeze-out. In this case, a portion of the electrons generated by impact ionization recombines with holes in the substrate, resulting in a smaller MW and a shorter retention time. As we showed in Figure 1d, electrons have to be injected into the region beneath the channel, so that they can modulate the potential of the channel. Basically, the capacitor associated with the substrate needs to be charged and since this capacitor scales proportional to the channel length, longer channel devices require more charges. As a result, a smaller MW for devices with longer gate is expected, which is confirmed in Figure 3c and Figure 3d (the blue lines). The MW can be enlarged by applying writing voltages simultaneously at both the source (V_SW_) and the drain (V_DW_), as seen in Figure 3c,d (the red lines): More charges are injected underneath the channel from both contacts, which can easily fulfill the requirement of state “1” as indicated in Figure 1. The writing causes an extremely small heat dissipation energy through the top transistor E_FET_. Here the E_FET_ is calculated by using the conventional method:^32,33^
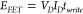
2

Unlike the conventional PDSOI 1T-DRAM the writing is performed at a large drain and gate voltage to enable band to band tunneling or impact ionization at the drain side, our device is written at V_G_ = 0 V (|V_G_| < |V_TH_|) and V_DW_ = -2 V. Thus, the current I_D_ is the off-current (∼10^–14^ A in Figure 3a). For a write time of 1 μs, *E*_*FET*_ = 2 × 10^–20^ = 20 zJ. This demonstrates that the transistor is not influenced by hot carriers generated through impact ionization in the substrate, owing to the isolation provided by the BOX layer, which is an important advantage over conventional 1T DRAM. However, measuring the impact ionization energy (or storage energy) presents a challenge with our current design. Based on the substrate current shown in Figure S4 measured from a large drain contact area (∼26 μm × 36 μm) at V_DW_ = 2.0 V and a 1 μs write time, the storage energy is around 40 fJ. This energy could be reduced by scaling down the drain contact area, as only regions near the channel affect memory behavior (Figure 1). Most of the contact area contributes minimally to memory operation but adds parasitic currents to the measured substrate currents. Additionally, thinning the BOX layer could exponentially boost the impact ionization rate and lower the write voltage. However, it would also linearly increase the capacitance, requiring an optimal BOX thickness for balanced performance. The reading energy consumption is in the order of 10 pJ, very similar to the conventional 1T DRAM memory. More details on the memory device characteristics with different gate lengths are presented in Figure S6.

The cycle-to-cycle variability of the C^2^RAM is characterized by cycling measurements of the I_D_–V_D_ characteristics at V_G_ = −0.6 V. Figure 3e showcases the cumulative probability distribution of memory characteristics derived from 15,000 I_D_–V_D_ measurement cycles at 5.5 K. During these measurements, V_D_ undergoes a forward sweep from 2.0 V (erase phase) to −2.5 V (write phase) and is then swept back to its starting point, as illustrated in Figure S7a. The “State 0” data is captured at V_D_ = −1.5 V, prior to reaching the write voltage of −2.5 V. Conversely, “State 1” is recorded at a voltage of −2.5 V following the writing process at the same voltage. The conductance of each state shows small variation. The current difference (ΔI_D_) between the backward and forward sweeping at a constant voltage V_D_ = −1.5 V is almost constant, showing a very small standard deviation of 4.56 μA, as indicated in Figure S7c. We also demonstrated small variabilities at other states as shown in Figure S7d.

The retention time is determined by measuring the drain current at a fixed V_G_ = −0.5 V and V_DR_ = −0.5 V, with hold-times ranging from 1 to 5400 s. These measurements are performed after writing at V_DW_ = −2.5 V or erasing at V_DE_ = 2.5 V. By fitting the retention data after writing, an extrapolation to 10 years is achieved, representing approximately 90% of ΔI_D_, as depicted in Figure 3f. This extrapolation demonstrates the nonvolatile property of C^2^RAM devices at 5.5 K.

We should point out that the performance of C2RAM is influenced by temperature. As the temperature increases, the memory window decreases, as illustrated in Figure 3b, which adversely impacts the retention time. However, in applications such as quantum computing, where the operating environment is maintained at a highly stable temperature, this effect is negligible. Measurements conducted at 77 K indicate some degradation in retention time; nevertheless, it remains above 10 years, as shown in Figure S8b. Therefore, the memory exhibits stability across a wide range of cryogenic temperatures below 77 K. Additional evaluations, which were performed after write/erase operations at a reduced voltage of 2.0 V, or after 10,000 write/erase cycles, further confirm robust retention time at 5.5 K, as depicted in Figure S8a and Figure S8d.

Neuromorphic computing devices operating at cryogenic temperatures offer promising solutions for qubit-related tasks, such as real-time quantum error correction decoding. These neural systems rely on nonvolatile analog memories to perform computational tasks.^34^ The multistate capability and nonvolatile nature of the C^2^RAM meet these specific requirements. Here, we demonstrate that a single C^2^RAM memory device can exhibit synapse properties, replicating the potentiation and depression behaviors observed in biological synapses.

The device showed already some neuron properties at 77 and 5.5 K when applying a long writing pulse (Figure S9). Here we present the potentiation and depression properties of a single C^2^RAM device as an artificial synapse, effectively mimicking the brain’s ability to store and erase memories. The measurement and the pulse configuration are schematically shown in Figure S10. Figure S11a–c presents the applied identical pulses. The gate voltage was maintained at −0.6 V. The reading currents were plotted as a function of the pulse number, as displayed in Figure 4a. At 5.5 K, both potentiation and depression characteristics were achieved with the writing and erase pulses. It is worth noting that the potentiation exhibited excellent linearity over 100 states (here the number of pulses), allowing for much faster, more stable and progressive, more energy-efficient learning. Conversely, the depression displayed a rapid decrease during the first few pulses. While potentiation is more crucial for learning processes, the improvement of depression can be achieved by modulating the erase pulse as illustrated in Figure S11d–f. The resulting long-term memory characteristics for the synapse are depicted in Figure S12a. Similar synapse plasticity was also obtained at 77 K (Figure S12b). This is of great significance as neuromorphic computing can be operated at 77 K with a significantly reduced required cooling power.

**Figure 4:**
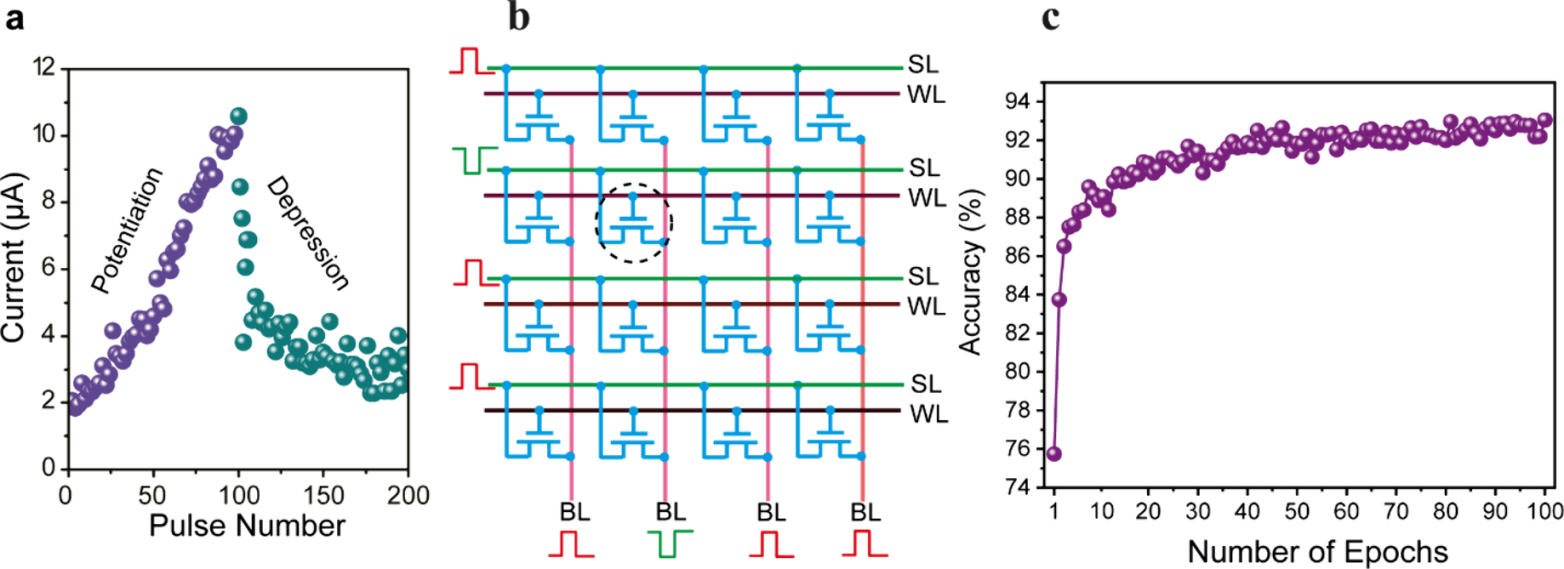
a. Long-term plasticity of the artificial synapse showing linear potentiation at 5.5 K. b. Configuration of 4 × 4 crossbar arrays. Only the device (marked with the black circle) with both negative voltages on the BL and SL can be written. c. Recognition accuracy of simulation based on the synaptic weight results in a. A high learning accuracy of 93% is achieved.

Leveraging the characteristics of the C^2^RAM device discussed earlier, we propose crossbar arrays for neuromorphic computing, exemplified by a 4 × 4 crossbar array with p-channel devices, as illustrated in Figure 4b. In this configuration, the word lines (WL) are connected to the gates, the bit lines (BL) to the drain contacts, and the source lines (SL) to the source contacts of the devices. Writing operations are performed by applying negative voltage pulses on the BL lines (Figure 4b). In this setup, the SL can act as the selector by applying a voltage <0 V during writing, as indicated in Figure 4b for the writing of the second row. Only the device with both negative SL and BL voltages can be written (see Figure S6). In other types of devices, such as memristive devices^35^ and FeFETs,^36^ an additional transistor is typically used as a selector for each memory device. However, our device technology eliminates the need for such additional transistors, enabling higher-density integration and lower write energy when writing simultaneously at the source and drain, as discussed previously.

We conducted a supervised learning simulation using a three-layer artificial neural network (ANN) as illustrated in Figure S13. As an illustrative example, we focused on recognizing a handwritten “0”, as shown in Figure S13. Remarkably, our simulation achieved an impressive recognition accuracy of 93% (Figure 4c) based on the measured results shown in Figure 4a. This significant result highlights the tremendous potential of C^2^RAM for enabling high-speed and low-power neuromorphic computing applications.

Compared to state-of-the-art devices such as memristive devices and FeFETs, C2RAM synapses exhibit a significantly higher number of potentiation states and greater potential for high integration density. Memristive devices,^35^ with two-terminal contacts, typically achieve fewer than 20 conductive states and are not suitable for cryogenic temperature application. Additionally, the crossbar structure used in memristors requires supplementary CMOS transistors as selectors, complicating integration. FeFET devices function similarly to transistors and can operate at low temperatures.^36^ However, FeFETs face scalability challenges due to the large variability in ferroelectric domains within the small area of a thin ferroelectric layer, leading to high device variability.

In summary, we have successfully demonstrated a single transistor memory cell C^2^RAM based on FDSOI technology. The memory device relies on the impact ionization and floating effect of the substrate at cryogenic temperatures. One notable feature of the C^2^RAM device is its ability to be written and erased to multiple states, which significantly increases the memory density. Furthermore, the retention time of the memory is remarkably long, reaching 10 years, making it highly suitable for nonvolatile storage applications. Another significant advantage of the C^2^RAM is its ultralow writing heat dissipation of 20 zeptojoules (zJ)/bit.

One particularly interesting aspect is the ability of the C^2^RAM to function as an analog memory for neuromorphic computing at cryogenic temperatures. The individual memory device exhibits excellent neuron-like behaviors, including integration, potentiation, and depression. The potentiation demonstrates perfect linearity with a large number of analog states, surpassing 100, which is advantageous for advanced neuromorphic computing applications.

The logic and analog capabilities of C^2^RAM, combined with the high scalability of FDSOI technology, offer significant potential for high-density memory applications at cryogenic temperatures. By integrating steep-slope FDSOI CMOS and silicon spin qubits, this technology enables the seamless integration of CMOS, memory, and silicon spin qubits on the same chip, paving the way for advanced cryogenic computing systems.

## Supporting Information Available

Additional experimental details, device fabrication process, device characterizations and artificial synapse measurements, including measurements methods (PDF)
