# Supplementary material for: An Energy Efficient Memory Cell for Quantum and Neuromorphic Computing at Low Temperatures
Source: Nano Lett. 2025 Apr 14;25(16):6374–81. doi: 10.1021/acs.nanolett.4c05855 (PMC12023033; doi:10.1021/acs.nanolett.4c05855)
Supplement: Supplementary file 1 — nl4c05855_si_001.pdf [file nl4c05855_si_001.pdf]

Supporting Information

An energy efficient memory cell for quantum and neuromorphic computing at low temperatures

Yi Han[1], Jingxuan Sun[1], Benjamin Richstein[2], Andreas Grenmyr [1,2], Jin Hee Bae[1], Frederic Allibert[3], Ionut Radu[3], Detlev Grützmacher[1], Joachim Knoch[2], Qing-Tai Zhao[1]*

[1]Institute of Semiconductor Nanoelectronics, Peter Grünberg Institute 9 (PGI 9) and JARA-Fundamentals of Future Information Technologies, Forschungszentrum Juelich, 52428, Germany.

[2]Institute of Semiconductor Electronics, RWTH Aachen University, 52056 Aachen, Germany.

[3]*SOITEC, 38190 Bernin, France.*

*Corresponding author. q.zhao@fz-juelich.de.

Materials and Methods

1. Fabrication process of C²RAM

The device is manufactured on ultra-thin body and ultra-thin buried oxide (UTBB) p-type (100) SOI substrates ($N_A = 1\times10^{15}$ cm^{-3}) with different gate lengths ranging from 100 nm down to 50 nm. The channel width is 800 nm. The fabrication process is illustrated in Fig.S1. The gate stack includes 5 nm HfO_2 deposited by atomic layer deposition (ALD) and 40 nm TiN by sputtering.

After patterning the gate stack with e-beam lithography, single crystalline $NiSi_2$ layers with smooth surface on source and drain are formed with an ultra-thin Ni layer by rapid thermal annealing (RTA) at 700 °C. The initial Si film at source and drain is fully silicided into $NiSi_2$ as shown in a cross-sectional transmission electron microscope (TEM) image (Fig. 2a). The single crystalline $NiSi_2$ layer at the source/drain is aligned to the TiN gate edge and shows a perfect interface to the BOX below. The $NiSi_2$/Si channel interface is on a (111) crystal facet because of the low formation energy of $NiSi_2$ on (111) surfaces. Then, B^+ implantations at tilted angles of 45° and 135° respectively are carried out followed by an RTA at 500 °C to drive-out and activate the implanted dopants. This process causes the formation of highly doped pockets close to the $NiSi_2$ edges at source and drain by dopant segregation[1,2]. The unreacted Ni is removed by selective chemical etching in diluted H_2SO_4 solution. Subsequently, a protective SiO_2 layer is deposited by plasma enhanced chemical vapor deposition (PECVD). Finally, contact windows for the source, drain, gate and back-gate are opened and metallized with Al using lift-off technology. The back-gate is located close to the channel by contacting the Si substrate through the BOX. No channel doping and no ground plane implants are used for these devices.

2. Measurement and characterization of C^2RAM

The device was characterized in a cryogenic probe station cooled with liquid helium. The data are measured using a Keithley CSC-4200 semiconductor analyzer with PMU and SMU connected to the cryogenic probe station.

3. The voltage configuration for writing

During the writing and erasing process, we set V_G to 0V and used a writing drain voltage (V_{DW}) below the threshold voltage (V_{TW}) for state "0" (Fig. S3b), and a $|V_{DW}| > |V_{TW}|$ for writing state "1" (Fig. 2b). The program time for writing and erasing was kept constant at 1ms for fast measurements.

During the hold time of 1s, V_G and V_D were both set to 0V. Reading out the state is performed by sweeping the gate voltage V_G from 1 V to -1 V at a constant reading drain voltage V_{DR} = -0.3 V to check the V_{TH} shift.

4. Characterization of the writing threshold voltage V_{TW}

The V_{WT} is characterized by the drain current I_D vs drain voltage V_D are shown in Fig. S3a at a gate voltage V_G = -0.6 V. V_D sweeps forward and backward between 1.0 V and -2.5V (here shows only the part at V_D < 0 V to focus on the memory effect). The erase part at V_D > 0 V can be found in Fig. S7a. A rapid increase of I_D occurs at around V_D = -1.9 V during the V_D forward sweeping from 1.0 V to -2.5 V. This voltage (V_D = -1.9 V) is defined as the writing threshold V_{TW}, where state "1" can be written at a $|V_D| > |V_{TW}|$. When the writing voltage is below the writing threshold V_{TW} (Fig. S3b), the reading I_D-V_G curve shows almost no shift compared to the reference one (Fig. S3c).

5. The writing speed

The writing and erasing speeds are evaluated by measuring the drain current (I_D) at reading voltages of V_{DR} = -0.1 V and V_G = -0.6 V while varying the writing/erasing time. Fig. S8c illustrates that I_D shows minimal changes for writing times ranging from 1 s to 1 μs. This behavior is also observed during the erase process. To further enhance the writing speed, a thinner BOX layer can be utilized, which exponentially increases the impact ionization rate as described in Eq. (1). However, employing a thinner BOX layer also increases the capacitance of the substrate, requiring a greater number of stored charges to modulate the channel potential. Achieving an optimal BOX layer thickness involves considering both the exponential increase of the impact ionization rate and the linear increase of the capacitance beneath the channel as the BOX layer thickness decreases.

6. The retention time

While the retention time is unaffected by a smaller writing voltage of V_{DW} = -2.0 V (Fig. S8a), it is influenced by temperature in a manner similar to the memory window. When the temperature increases to 77 K, ΔI_D decreases over time; however, approximately 50% of ΔI_D is still retained after 10 years, as indicated in Fig. S8b, much longer than the recent published memory with SiGe floating body at 77 K (8000 s) [3].

7. The neuromorphic characterization of C^2RAM

In Fig. S9a, the response current to a long writing pulse applied at the drain electrode at 5.5 K is presented. The current measurement was performed at the source contact of the device. We should point out, different from the results shown in Fig. S8 where the current is measured by reading, here the current is measured during writing at a constant gate voltage. Over time, the current gradually increases, displaying an S-shaped curve, which is a characteristic sigmoid function commonly observed in learning curves within neural networks. This behavior is similarly observed at 77 K, as depicted in Fig. S9b. These figures also demonstrate the neuronal integrate-and-fire function. As discussed in Fig. 1, the memory functionality relies on impact ionization at the BOX/silicon substrate interface, which occurs rapidly[4]. However, electrons generated by impact ionization near the drain side must charge the capacitor beneath the channel to reduce the threshold voltage of the transistor. Consequently, the gradual accumulation of electrons in the capacitor leads to a gradual reduction of the threshold voltage over time, resulting in a corresponding increase in the current.

We applied a series of repeated identical writing pulses to the drain of the C^2RAM device. These pulses had an amplitude of -2.5 V, a pulse width of 100 ns, and an interval of 100 ns, as illustrated in the inset of Fig. S9c. The measured source current, taken at a fixed V_G = -0.6 V, is presented as

a function of the pulse number in Fig. S9c, highlighting the long-term memory capability of the artificial synapse. The observed good linearity and the requirement for a large number of pulses are crucial for achieving high-accuracy learning.

Furthermore, similar synapse plasticity was also obtained at 77 K as shown in Fig. S12b. We should point out that we observed asymmetric potentiation and depression characteristics under our experimental conditions. Specifically, potentiation exhibited good linearity and a greater number of states, while depression demonstrated poorer linearity and fewer states. The good linearity and the large number of states in potentiation allow for much faster, more stable and progressive, more energy-efficient learning. We are further characterizing our devices by fine-tuning the reading and writing conditions as indicated in Fig. S11d-f, particularly focusing on improving the linearity of depression as shown in Fig. S12a.

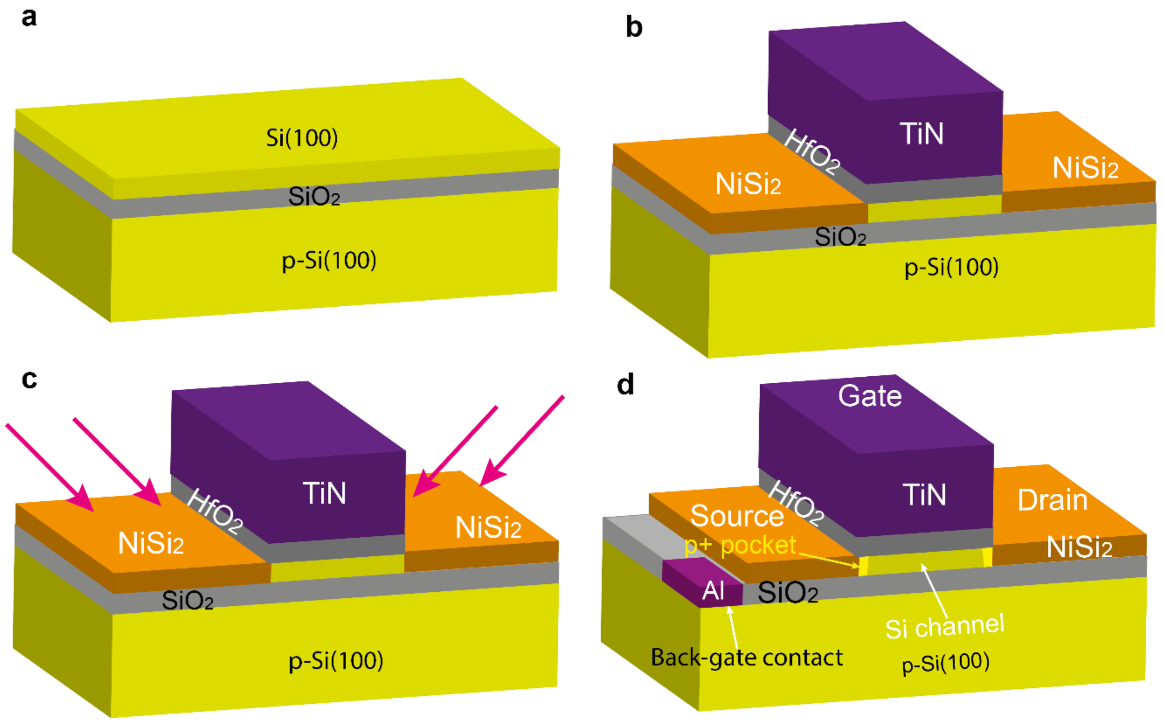

Figure S1. Device fabrication process. a. UTBB SOI wafer as the starting substrate. **b**. Device structure after gate formation and silicidation at source/drain. The source/drain is fully silicided into $NiSi_2$. **c**. Tilted B^+ ion implantation into $NiSi_2$. **d**. Highly doped pockets formed at the $NiSi_2$/Si channel interfaces at both the source and drain after annealing. Aluminum (Al) back-contact is formed by etching through the BOX layer.

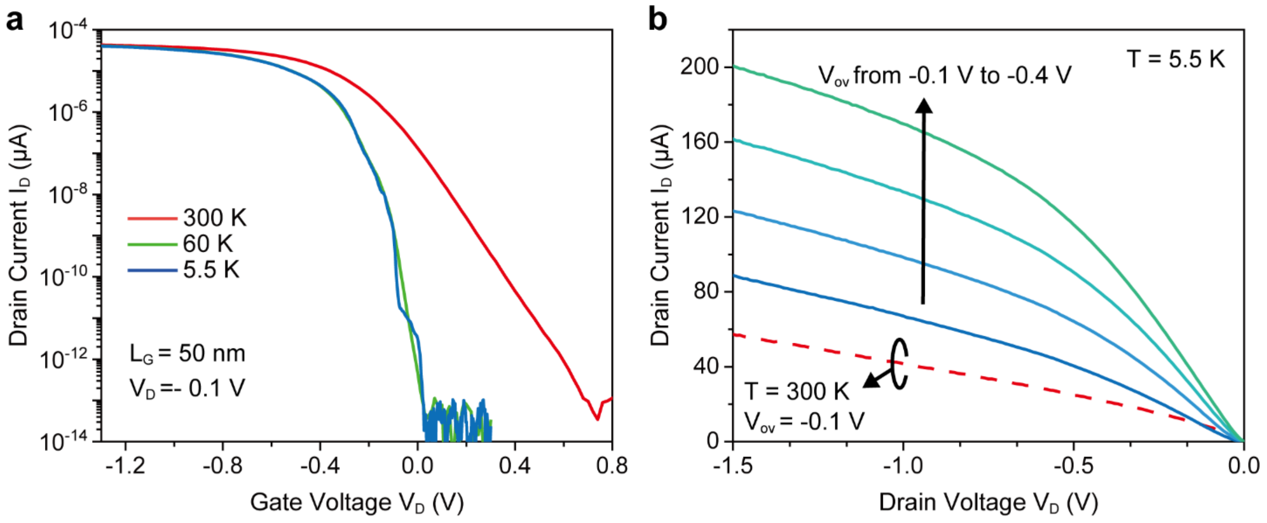

Figure S2. Device characterization at temperatures from 300 K to 5.5 K. a. I_D-V_G transfer characteristics for the fabricated device with a gate length of L_g=50 nm measured at temperatures of 300 K, 60 K, and 5.5 K, showing reduced subthreshold swing SS with reducing temperature. **b**. I_D-V_{DS} output characteristics measured at 5.5 K and overdrive voltages ($V_{ov} = V_G - V_{TH}$) from -0.1 V to -0.4 V. The dashed red curve presents the I_D-V_{DS} output characteristics measured at 300 K and V_{ov} = -0.1 V, as a reference. The comparison to the 300 K results indicates a large improvement of drain currents at 5.5 K due to the mobility improvement.

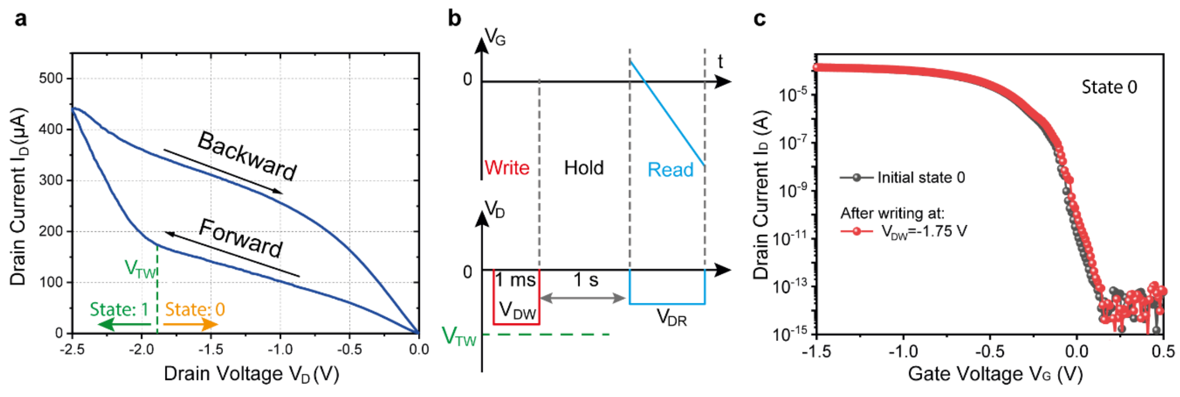

Figure S3. The characterization of the V_{TW}. a. I_D-V_D characteristics at V_G = -0.6 V showing a rapid increase of the drain current I_D at $|V_D| > |V_{TW}|$ and a clockwise hysteresis, where V_{TW}=-1.75 V. **b**. Voltage configuration for writing, holding and reading state "0" with a low writing voltage $|V_{DW}| < |V_{TW}|$. **c**. Reading of state "0" before and after writing with V_{DW} = -1.75 V, showing unchanged states.

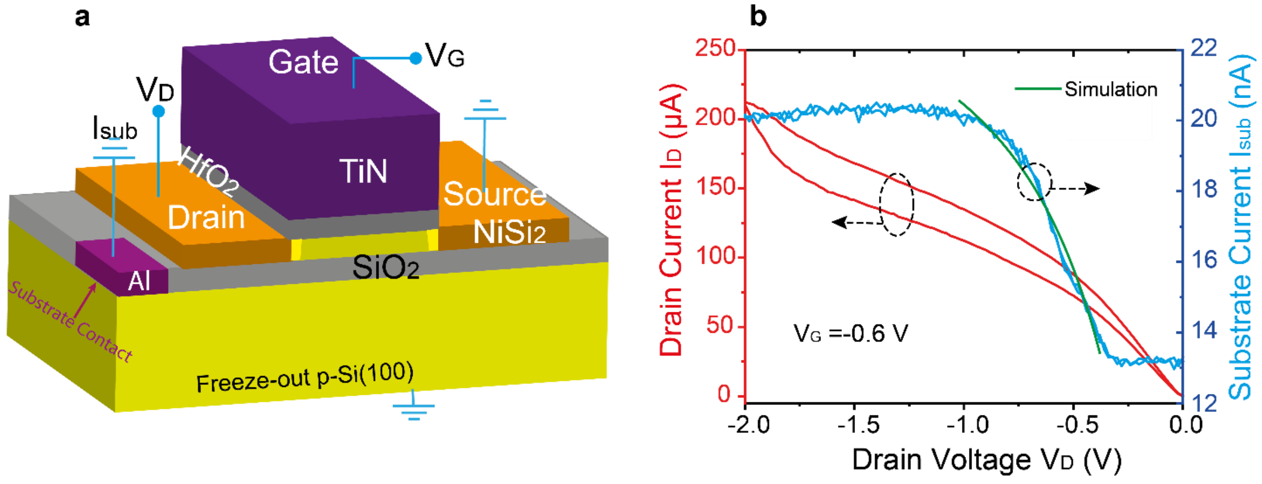

Figure S4. **Measurement of the substrate current. a**. Schematic showing the configuration of the device for the substrate current measurement using a substrate contact of aluminum (Al) through the BOX layer without applying any bias. **b**. The measured substrate currents I_{sub} and drain currents I_D as a function of the drain voltage V_D. The green curve is the fitting of the substrate current using the impact ionization theory. I_{sub} is caused by the holes. These results confirm that the impact ionization induced holes dissolve to the substrate, leaving electrons staying in the region below the channel.

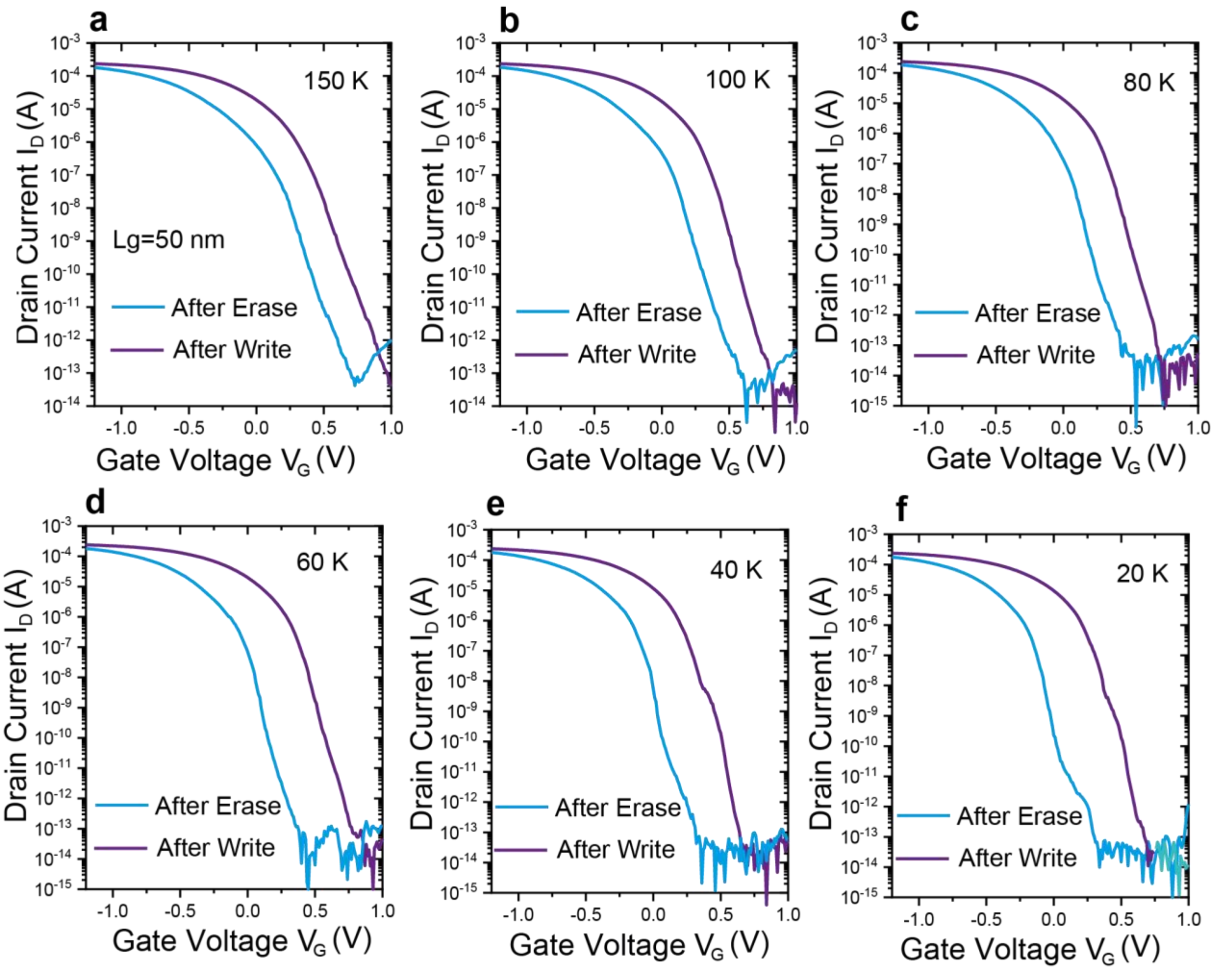

Figure S5. Memory dependence on temperature. I_D-V_G transfer characteristics after writing and erase at the same conditions for the fabricated device with a gate length of L_g = 50 nm measured at temperatures from 150 K to 20 K, showing the increasing memory window with cooling of the device.

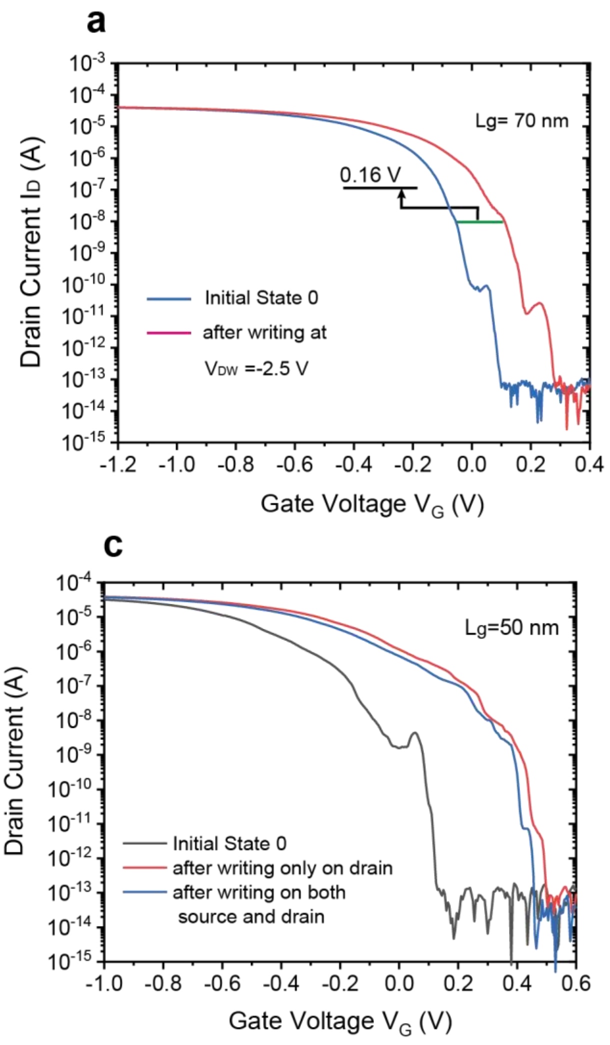

Figure S6. Characteristics for different gate length transistors under various writing conditions at 5.5 K. **a**. A 70 nm gate length transistor after writing at V_{DW} = -2.5 V, showing a memory window of 0.16 V. **b**. A gate length L_g=100 nm device after writing only on the drain at V_{DW} = -2.5 V, showing almost no writing. The writing simultaneously at both the source and drain with writing voltages V_{DW} =V_{SW} =-2.5 V can realize state "1". **c**. For the short channel device with L_g = 50 nm, the writing only on the drain with V_{DW} = -2.0 V showing similar writing window with the writing on both the source and drain simultaneously with V_{DW} = V_{SW} = -2.0 V. **d**. Applying a negative voltage on the drain V_{DW} = -1.4 V and a positive voltage on the source V_{SW} = 2.0 V cannot write state "1". When a reverse voltage on the source side is applied during the writing process on

the drain side, electrons induced from the drain side recombine with holes induced from the source side, resulting in a reduced number of carriers stored underneath the channel. Therefore, the source can be used as a selector for writing in short channel devices.

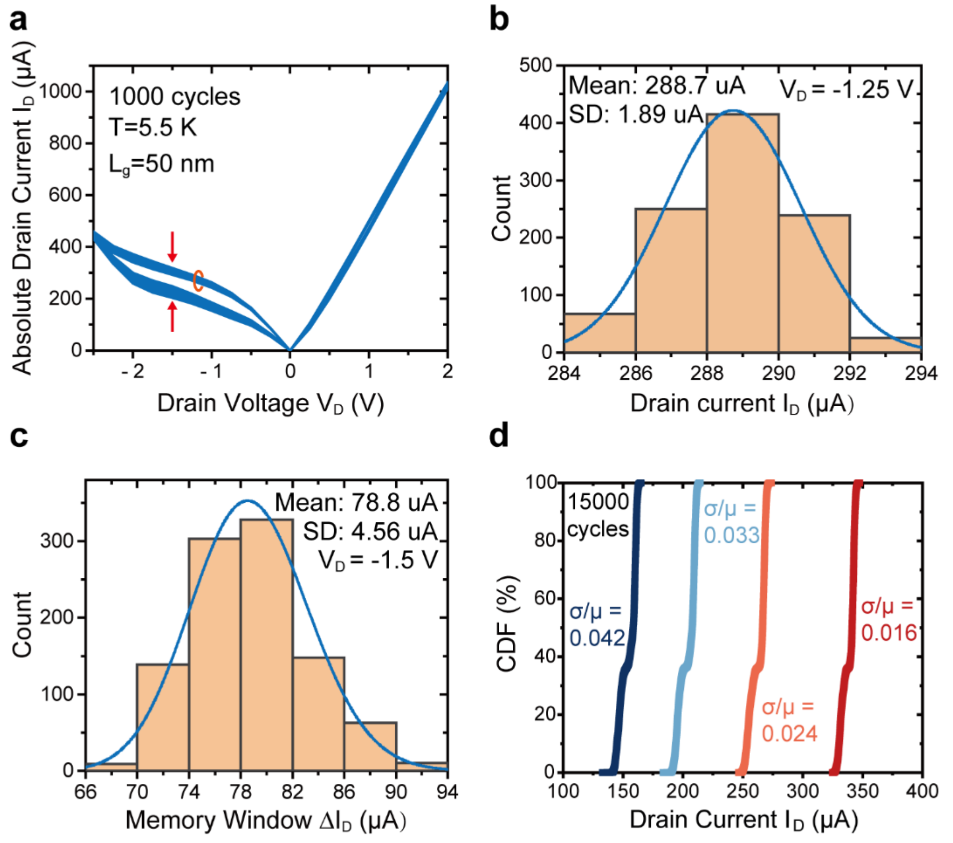

Figure S7. Variability measurements. **a**. 1000 circles I_D-V_D characteristics measurements showing a very small variation of both the currents and current difference. **b**. Variation of the drain current measured at V_D = -1.25 V in the backward direction as marked by the red circle in (a), showing a small standard deviation of 1.89 μA. **c.** The current difference (ΔI_D) between the backward and forward sweeping at a constant voltage V_D = -1.5 V as marked by red arrows in (a), showing a mean value of 78.8 μA and a small standard deviation of 4.56 μA. **d.** Cumulative probability distribution of multi-states showing very small variation, demonstrating high reliability.

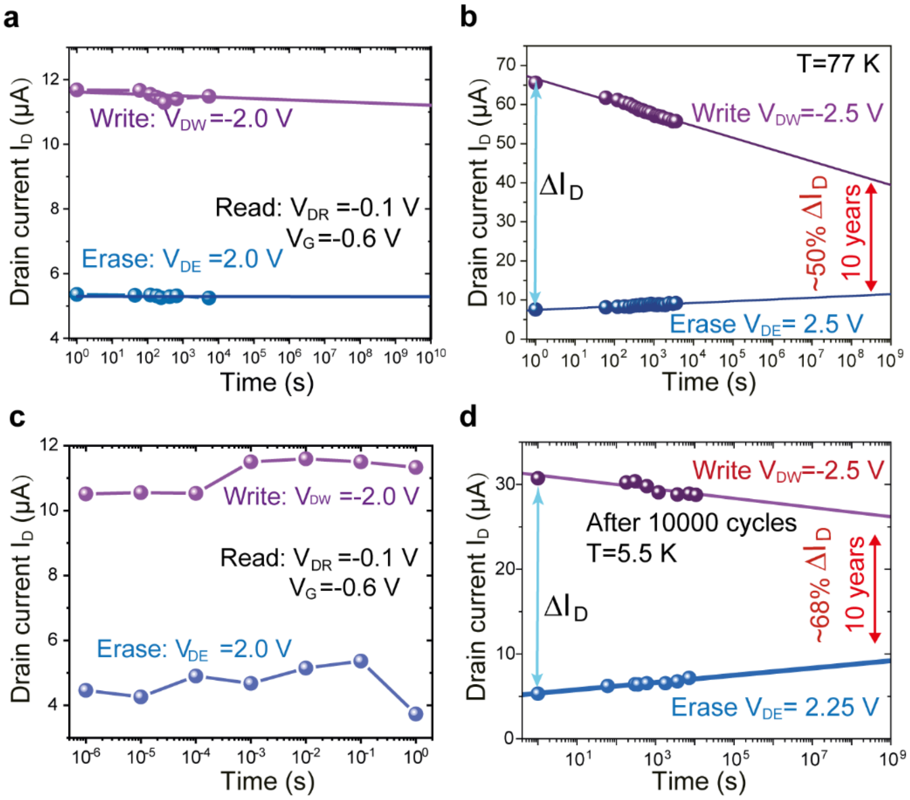

Figure S8. Retention and writing time measurements. **a**. Retention time measured at a writing voltage V_{DW} = -2.0V and an erase voltage V_{DE} =2.0 V, at 5.5K, showing a high retention time. **b**. The retention time is shorter at 77K compared to that measured at 5.5K. **c**. The writing and erase speed demonstrated by measuring the reading current after 1s write/erase. The reading current I_D shows minimal changes for writing/erase times ranging from 1μs to 1s. **d.** Retention time measured after 10000 writing/erasing cycles at 5.5K. V_{DR}=-0.3 V and V_{GR}=-0.5 V.

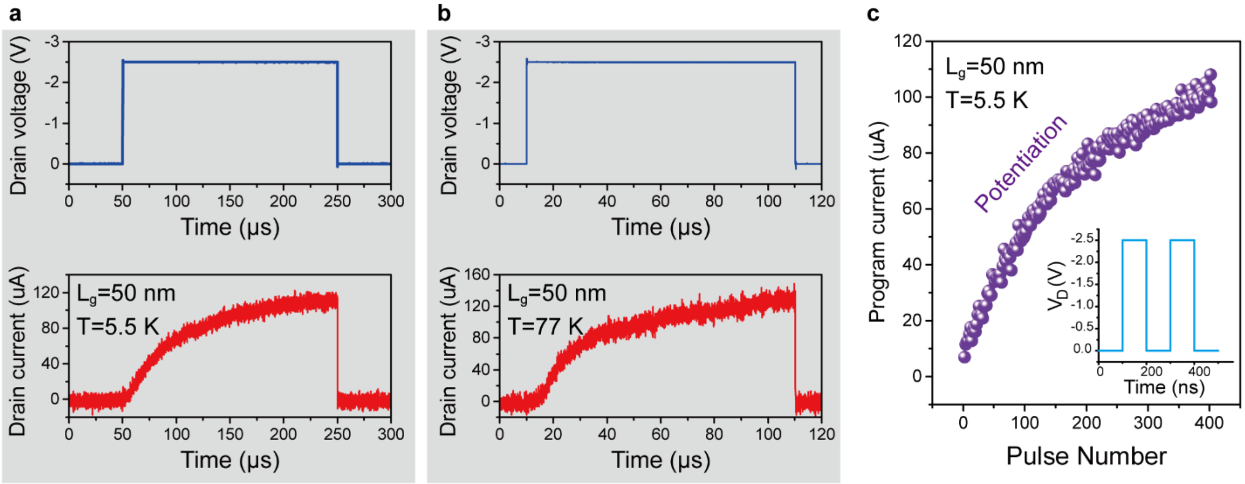

Figure S9. The neuromorphic characterization of C^{2}RAM. a. and **b.** Applying a long write voltage pulse (top) on the drain the response drain current shows neural sigmoid integration function at 5.5 K and 77 K. **c.** Measured current as a function of the writing pulse number, showing potentiation synaptic characteristics. The inset displays the pulse sequence.

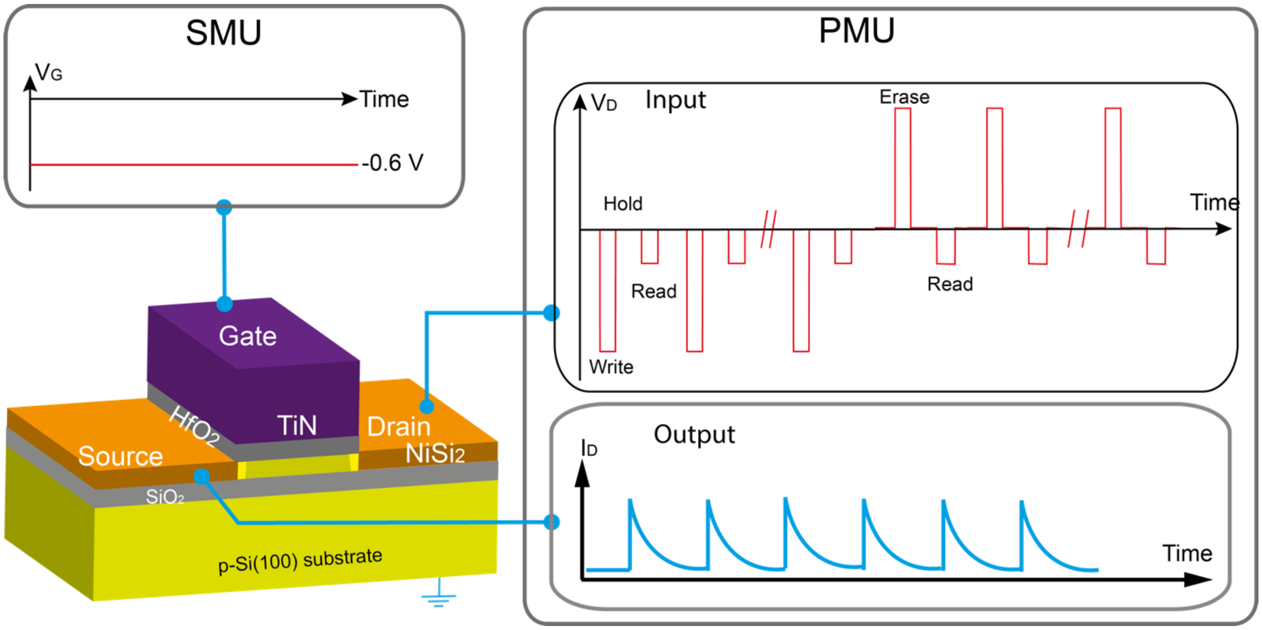

Figure S10. Artificial synapses characterization setup. The setup including a digital oscilloscope, a high speed trans-impedance amplifier and an arbitrary waveform generator. The waveform for the input signals on gate and drain are schematically shown.

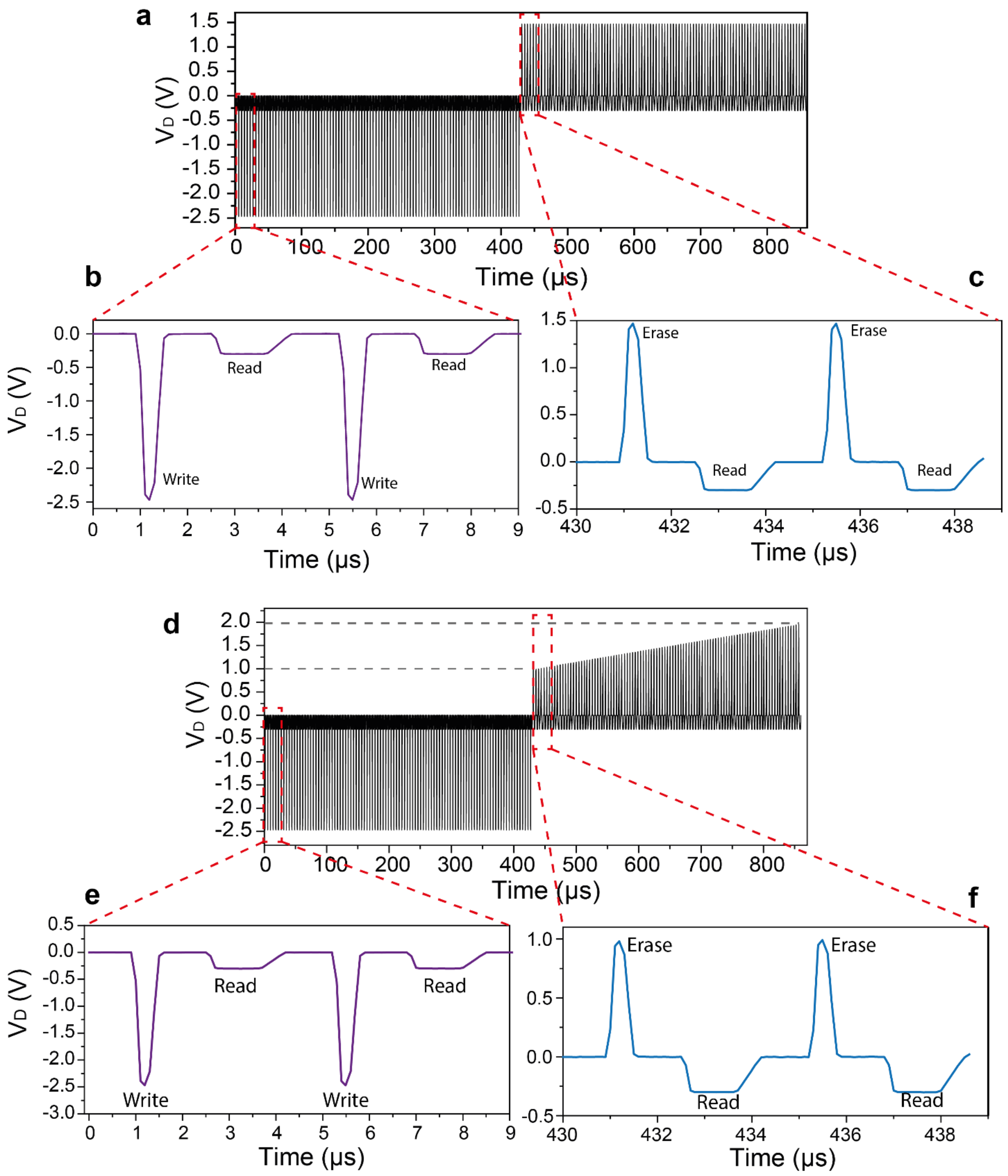

Figure S11. **The pulse sequence.** Voltage pulses applied on the drain for artificial synapse characterization with identical pulses for both potentiation and depression (a-c), with a voltage amplitude of -2.5 V and a duration of 0.5 µs followed by a reading pulse of -0.3 V after hold time of 1 µs. After 100 repeated writing/reading pulses the erase pulse was triggered, which has an

amplitude of 1.5 V and pulse duration of 0.5μs. This was followed by a reading pulse of -0.3 V after 1μs holding time. (d)-(f) show non-identical pulses for depression. The erase pulses increase with pulse number using $V_{DE} = 1.0 + 0.01n$, where n represents the erase pulse number ranging from 0 to 100.

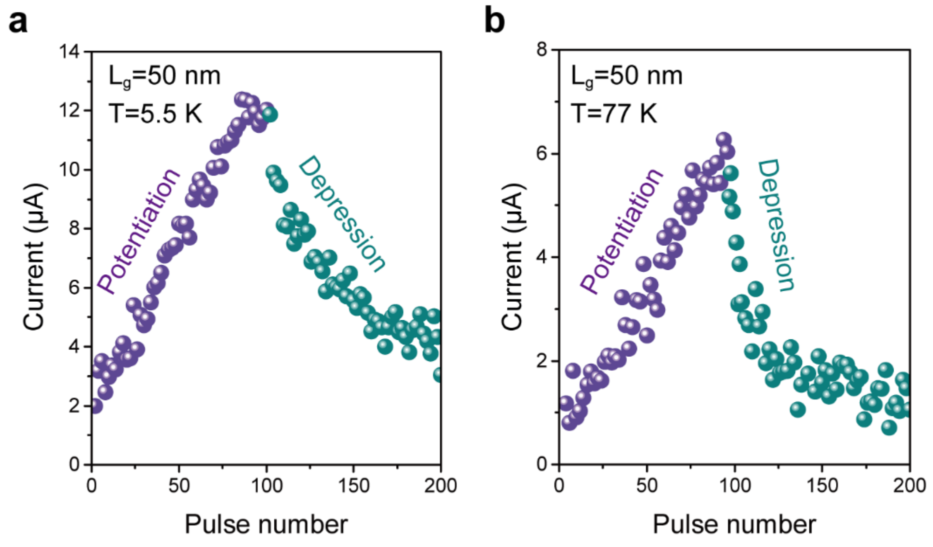

Figure S12. **Artificial synapse characteristics. a.** Long-term plasticity of the artificial synapse showing improved depression by using non-identical pulses as shown in Figure S11d-f at 5.5 K. **b.** Long-term plasticity of the artificial synapse showing linear potentiation at 77 K, similar to that shown in Fig.4a at 5.5 K.

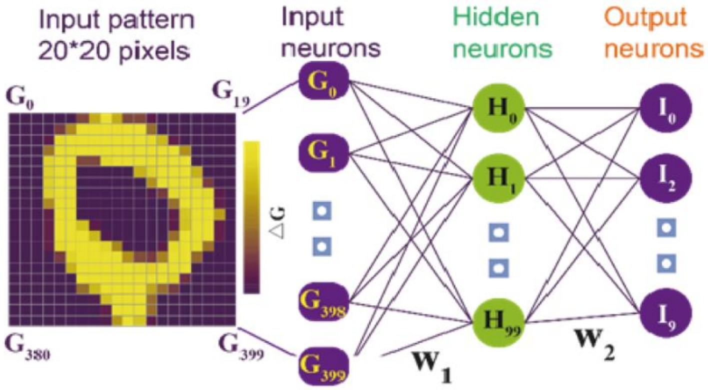

Figure S13: **The ANN simulation.** Schematic diagram of the three-layer perceptron with 400 input neurons, 100 hidden layer neurons, and 10 output neurons based ANN simulation. An input pattern of handwritten "0" is used as the input.

References.

1. Han, Y.; Sun, J.; Richstein, B.; Allibert, F.; Radu, I.; Bae, J.-H.; Grutzmacher, D.; Knoch, J.; Zhao, Q.-T. Steep Switching Si Nanowire P-FETs With Dopant Segregated Silicide Source/Drain at Cryogenic Temperature. *IEEE Electron Device Lett.* **2022**, *43* (8), 1187–1190. https://doi.org/10.1109/LED.2022.3185781.

2. Han, Y.; Xi, F.; Allibert, F.; Radu, I.; Prucnal, S.; Bae, J.-H.; Hoffmann-Eifert, S.; Knoch, J.; Grützmacher, D.; Zhao, Q.-T. Characterization of Fully Silicided Source/Drain SOI UTBB NMOSFETs at Cryogenic Temperatures. *Solid. State. Electron.* **2022**, *192*, 108263. https://doi.org/10.1016/j.sse.2022.108263.

3. Chakraborty, W.; Shrestha, P.; Gupta, A.; Saligram, R.; Spetalnick, S.; Campbell, J.; Raychowdhury, A.; Datta, S. Multi-Bit per-Cell 1T SiGe Floating Body RAM for Cache Memory in Cryogenic Computing. In *2022 IEEE Symposium on VLSI Technology and Circuits (VLSI Technology and Circuits)*; IEEE, 2022; pp 302–303. https://doi.org/10.1109/VLSITechnologyandCir46769.2022.9830483.

4. Tarekegne, A. T.; Hirori, H.; Tanaka, K.; Iwaszczuk, K.; Jepsen, P. U. Impact Ionization Dynamics in Silicon by MV/Cm THz Fields. *New J. Phys.* **2017**, *19*, 123018. https://doi.org/10.1088/1367-2630/aa936b.
